# *Trichuris* spp. in Animals, with Specific Reference to Neo-Tropical Rodents

**DOI:** 10.3390/vetsci8020015

**Published:** 2021-01-21

**Authors:** Kegan Romelle Jones

**Affiliations:** 1 Department of Basic Veterinary Sciences (DBVS), School of Veterinary Medicine (SVM), Faculty of Medical Sciences (FMS), University of the West Indies (UWI), Mt. Hope Campus, Trinidad and Tobago; keganjones11@gmail.com; 2 Department of Food Production (DFP), Faculty of Food and Agriculture (FFA), University of the West Indies (UWI), St. Augustine Campus, Trinidad and Tobago

**Keywords:** agouti, lappe, capybara, guinea pig, *Dasyprocta leporina*, *Agouti paca*, *Cuniculus paca*, *Hydrochoerus hydrochaeris*, *Trichuris*

## Abstract

Trichuriasis is the clinical disease of animals infected with the parasite of the genus *Trichuris*. This review attempts to present information on *Trichuris* spp. infestation in neo-tropical rodents that are utilized for meat consumption by humans. Neo-tropical rodents utilized for meat production can be divided into two categories: those that have been domesticated, which include the guinea pig (*Cavia porcellus*), and those that are on the verge of domestication, such as the capybara (*Hydrochoerus hydrochaeris*), lappe (*Cuniculus paca*/*Agouti paca*), and agouti (*Dasyprocta leporina*). This document reviews the literature on the species of *Trichuris* that affects the rodents mentioned above, as well as the clinical signs observed. The literature obtained spans over sixty years, from 1951 to 2020. *Trichuris* spp. was found in these neo-tropical rodents mentioned. However, there is a dearth of information on the species of *Trichuris* that parasitize these animals. The capybara was the only rodent where some molecular techniques were used to identify a new species named *T. cutillasae*. In most cases, *Trichuris* spp. was found in combination with other endoparasites, and was found at a low prevalence in the lappe and guinea pig. The presence of *Trichuris* spp. ranged from 4.62–53.85% in the agouti, 4.21–10.00% in the lappe, 50% in the capybaras, and 1–31% in guinea pigs. Further work must be done towards molecular identification of various *Trichuris* spp. present in these rodents, as well as the clinical effect of infection on the performance of agouti, lappe, capybara, and guinea pigs.

## 1. Introduction

**Figure 1:**
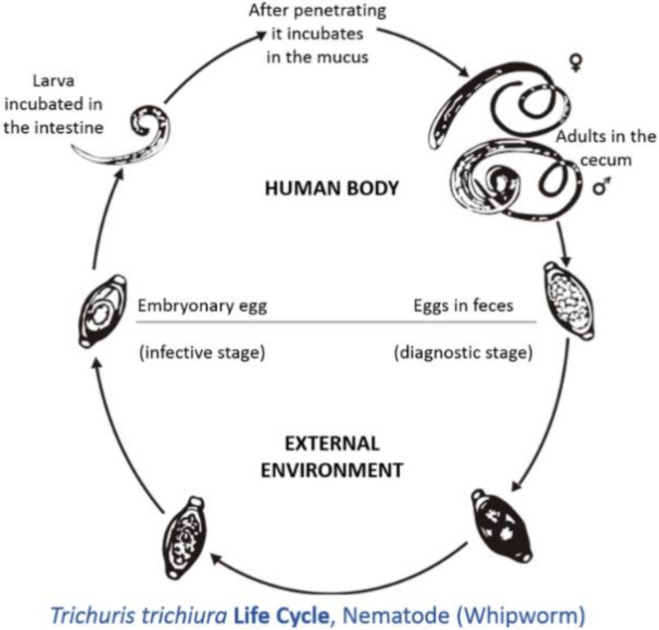
Life cycle of *Tichuris trichiura* (taken from [17]).

The neo-tropics is a geographical region located in the western hemisphere between the Tropic of Cancer and the Tropic of Capricorn. Geographical territories present within this zone include the southern parts of North America, all of Central America, the northern parts of South America, and all of the Caribbean [1]. Animals that are present in this region can be categorized into three groups: imported domesticated animals [2], domesticated animals originating from the neo-tropics [3], and non-domesticated neo-tropical animals [4]. For the purpose of this review, neo-tropical rodents that are included belong to the domesticated and non-domesticated groups. Domesticated neo-tropical rodents, such as the guinea pig, are utilized in South America for their meat and are reared in captivity to provide meat protein for rural villages. The guinea pig is able to utilize household waste and provide income and food for these communities [5,6]. Neo-tropical rodents on the verge of domestication are the agouti, lappe, and capybara. These animals have been reared in captivity in South America and the Caribbean for their meat [1]. These animals have been able to breed in captivity: the agouti produces four offspring per year [7], the lappe produces two offspring per year [8], and the capybara can produce eight offspring per year [9,10]. These animals are ideal in that they can utilize local feed resources and are adapted to local conditions of high heat and humidity. The meats produced by these rodents are highly nutritious, with high protein values and low fat and cholesterol concentration [11,12,13,14]. *Trichuris* spp., also known as whipworms, have parasitized many domesticated species, causing enteritis, diarrhea, and weight loss [15]. *Trichuris* spp. adults live in the caecum and colon; this predilection site has occurred due to evolution. The life cycle is direct; eggs with characteristic bi-polar plugs are passed in the feces and take two to three weeks to become infective (Figure 1) [16]. Animals become infected by the ingestion of infective eggs [16]. However, there has been limited information on the effects of *Trichuris* spp. on neotropical rodents (domestic and semi-domestic). Thus, the objective of this review is to summarize the species of *Trichuris* that parasitizes these rodents, the effect of this parasite on these animals, and the zoonotic potential of this pathogen.

## 2. Methodology

For the purpose of this review, reports and articles were searched for in scholarly publication databases (Google Scholar, PubMed, and UWI linc). Search terms used were specific species names (e.g., rodents, guinea pig (*Cavia porcellus*), agouti (*Dasyprocta leporina*), capybrara (*Hydrochoerus hydrochaeris*), and lappe (*Cuniculus paca*/ *Agouti paca*)) combined with the term “*Trichuris”* or “trichuriasis”. Searches were conducted for articles from 1990 to November 2020. Approximately 220 articles were identified for the review, but only 101 were appropriate to be included in the final manuscript. All sources were assessed by the author for relevance, credibility, and scientific inclusion, to ensure the thoroughness and accuracy of review. 

## 3. *Trichuris* spp. of Veterinary and Public Health Importance

### 3.1. Trichuriasis of Man

Trichuriasis is one of the major infectious diseases of children in developing countries [18]. *Trichuris trichiura* is a major, soil-transmitted helminth targeted by the World Health Organization in their mass drug administration program for pre-school and primary school children in endemic developing countries [18]. There have been several cases of trichuriasis reported in humans. In some cases, it has been due to three *Trichuris* spp.: *T. trichiura*, *T. vulpis*, and *T. suis*. Humans have been infected with *T. vulpis*, and the diagnosis was made based on the morphology of the eggs and vulva from an adult female [19]. Molecular techniques were used on *Trichuris* spp. egg present in feces to identify *T. suis* and *T. trichiura* in human populations from Thailand [20]. *T. suis* has been experimentally given to humans, and the author stated that feces were negative for *Trichuris* eggs 40 days post-infection [21]. Experimentally treated patients showed no symptoms of gastrointestinal distress [21]. In contrast to the previous studies, Kradin et al. [22] showed that iatrogenic infection with *T. suis* resulted in a persistent active infection in man. Pathological findings from colonic biopsies showed several round helminths beneath the ileocecal mucosa epithelium [22]. 

*Trichuris trichiura* has human and non-human primates as its natural hosts [23]. Mixed infections with various *Trichuris* spp. in humans have been documented. There have been cases of mixed infections with *T. vulpis* and *T. trichiura* [24,25]. The identification of the species of *Trichuris* spp. was based on the morphology of eggs [24] and polymerase chain reactions of the helminth eggs [25]. *Trichuris trichiura* and *T. vulpis* was also found in the stool samples of dogs that roamed around the community. This shows that dogs are key to the transmission of *Trichuris* spp. to humans, but further work needs to be done to validate this finding [25]. 

Infections with *T. vulpis* have been reported in children and adults [19,26,27]. However, all cases of trichuriasis in humans caused by *T. vulpis* have had some association with dogs, and the diagnosis was made based on morphology of eggs present in the feces. Clinical signs reported in humans are abdominal discomfort, epigastric pain, nausea, vomiting, diarrhea, and poor appetite [24]. Patients with *T. vulpis* [24,26,27] and *T. trichiura* [19] have been treated with mebendazole and albendazole with improvements of clinical signs [19,24,26,27]. However, in vivo studies on albendazole and mebendazole have shown little efficacy against *T. trichiura* [28]. At 14 days post-treatment, there was no difference in the disease prevalence seen between treatments of patients with 400 grams of albendazole [28]. Therefore, alternative anthelmintic treatment against *T. trichiura* should be investigated. Ivermectin has been used to treat *Trichuris* spp.; however, it is very ineffective, as these parasites have become resistant to this drug. However, due to the increased prevalence of anthelmintic resistance, the drugs used to treat trichuriasis should be done with caution.

### 3.2. Morphological and Molecular Identifications of Trichuris spp.

#### 3.2.1. Morphological Identification of *Trichuris* spp. in Pigs, Dogs, Cats, Humans, and Non-Human Primates

Morphological analysis of *Trichuris* spp. has been used for identification within various host species. *Trichuris trichiura* infection has been investigated in humans, non-human primates, and pigs, but based on morphological analysis, the *T. trichiura* found in humans and non-human primates were indistinguishable [29]. In pigs, *T. suis* was differentiated from *T. trichiura*, based on the lack of peri-cloacal papillae in adult specimens. In female specimens, there were no morphological differentiation between *T. suis* and *T. trichiura* [29]. Ruminants evaluated in India using morphological analysis identified *T. ovis* as the major parasite [30]. 

Further research was done in domestic cats in St. Kitts. Based on the size of the *Trichuris* spp. identified, authors believed that it was *T. campanula*, but based on the vulva structure the authors confirmed it was *T. serrata*. In conclusion, the authors, identified the parasite as *T. serrata*, but recommended that molecular studies must be done in order to reliably identify this parasite [31]. In dogs, male and female adult *T. vulpis* could be identified based on nine parameters (including body length, length of cuticular processes, and width of body at tail part) [32]. Male *T. vulpis* can be distinguished from other species by spicule sheath ornamentation (the dimensions of the spicule) [32]. 

Recently, the morphometric approach analyzing the adult worms and eggs of *Trichuris* spp. of non-human primates were analyzed [33,34]. Morphometric data on the adult worms showed that features present in the females made them indistinguishable for species characteristics, but adult male worms may be used to differentiate *Trichuris* populations [33]. Geometric morphometric analysis is a new diagnostic tool that can be used to differential *Trichuris* spp. present in non-human primates. However, further data must be collected to determine the sensitivity and specificity of this diagnostic tool [34]. Combination of various techniques, such as the use of molecular and morphological analysis, should be performed for confirmation of various *Trichuris* spp. [33].

#### 3.2.2. Molecular Identification of *Trichuris* spp. in Domestic and Non-Domestic Ruminants

Molecular techniques have been used to identify various *Trichuris* spp. in their animals or human hosts. Such techniques have been applied to *Trichuris* spp. found in ruminants (both domesticated and non-domesticated). Four *Trichuris* spp.—*T. discolor*, *T. ovis*, *T. globulosa* and *T. skrjabini*—have been identified as inhabiting the caecum and colon of ruminants [35,36,37,38,39,40,41,42,43,44,45]. One of the major discoveries was the identification of *T. globulosa* and *T. ovis* as the same species by isoenzymes [35], using second, internally transcribed spacer ribosomal DNA (ITS2 rDNA) [38] and ITS1-5.8S-1TS2 [37]. Further molecular analysis was done comparing *T. ovis* and *T. discolor*, where the entire mitochondrial DNA (mtDNA) was analyzed [42], and with the use of internally transcribed spacers 1, 2, and 16S, partial DNA sequencing (ITS1, 2, 16rDNA) was completed [44]. Based on mtDNA and rDNA, *T. ovis* and *T. discolor* can be classified as two different species. 

*Trichuris skrjabini*, found in small ruminants (sheep and goats), was characterized using isoenzymes [36], ITS1-5.8S-1TS2 [37], and cytochrome oxidase subunit 1 and mitochondrial 16S rDNA [39]. Authors have stated that *T. skrjabini* is an independent species but has close relations to other *Trichuris* spp. that parasitize small ruminants. *Trichuris discolor* has been identified in domestic ruminants with the use of molecular techniques; however, it was recently identified in wild ruminants, such as the roe deer (*Capreolus capreolus*), sika deer, (*Cervus nippon*), red deer (*Cervus elephus*), fallow deer (*Dama dama*), and mouflons (*Ovis orientalis musimon*) [43,44,45]. In wild ruminants, *T. discolor* was identified with use of ITS1-5.8S-1TS2 [43,44,45], but in cattle different populations of *T. discolor* in Iran, Spain, and Japan were investigated using 16S partial gene mtDNA, as well as ITS1 and 2 [43]. Callejon et al. [43] noted that there were specific populations of *T. discolor* groups based on geographical location. The author noted that one reason may be due to two cryptic species of *T. discolor* from Japan and Iran, as well as another from Spain. 

#### 3.2.3. Molecular Identification of *Trichuris* spp. in Cats, Dogs, Pigs, Humans, and Non-Human Primates 

*Trichuris* spp. has also been identified molecularly in pets, such as dogs and cats. In cats it is associated with typhlitis, which also occurs in other animals [46]. Identification of *T. serrata* (cats) and *Trichuris vulpis* (dogs) was accomplished through the use of 18S rDNA (cats) and enzyme-linked immunosorbent assay (ELISA) and ITS1-5.8S-1TS2 (dogs) [47,48,49]. Comparative genetic studies were done of the *T. vulpis* found in dogs and *T. suis* found in pigs (wild and domesticated). There was a difference seen in amplified ITS1-5.8S-1TS2 rDNA between the *T. vulpis* found in dogs and *T. suis* found in pigs. Interestingly, *T. suis* collected from wild pigs (*Sus scrofa scrofa*) and domestic pigs (*Sus scrofa domestica*) showed no sequential genetic differences [49].

Several non-morphological processes were used to identify *T. suis* found in pigs using isoenzymes [50], ITS 1 and ITS2 regions of rDNA [51], large mitochondrial subunits and ITS2 [52], and nuclear ribosomes (18S, ITS2) [18]. Due to the zoonotic potential of *T. suis* and its morphological similarity to *T. trichiura* previous molecular studies have been done in both human and non-human primates [53,54,55]. *Trichuris* spp. was taken from pigs (wild and domestic) and non-human primates (*Colobus guereza kikuyensis* and *Nomascus gabriellae*) and analyzed by amplification of rDNA (ITS1-5.8S-1TS2). The authors confirmed that the *T. suis* found in pigs was genetically different from *T. trichiura* in *Colobus guereza kikuyensis* and *Nomascus gabriellae* [53]. Nissen et al. [54] conducted a similar study to Cutillas et al. [53], but *T. suis* and *T. trichiura* were identified in pigs and humans in Uganda. The gastrointestinal tract of pigs only contained *T. suis*, while in humans *T. trichiura*, *T. suis*, and a heterozygous type was identified [54]. This showed that the use of ITS 2 and β-tubulin allowed the identity of several species of *Trichuris* in humans to be highlighted.

**Figure 2:**
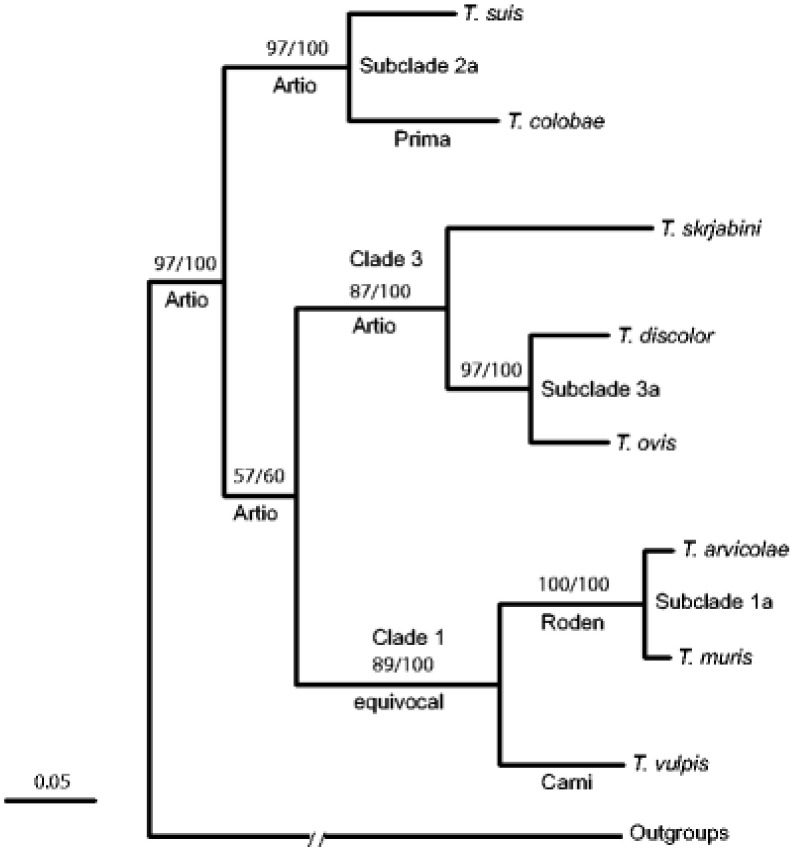
Phylogenic tree of *Trichuris* spp. (taken from Cutillas et al. [53]).

The research done by Cutillas et al. [53] and Nissen et al. [54] highlights the fact that humans and non-human primates may be infected with several species of *Trichuris* that are generally classified as *T. trichiura.* This was seen with *Trichuris* spp. samples taken from the wild Japanese macaques (*Macaca fuscata*), where the *Trichuris* spp. identified had genetic (18S rDNA) dissimilarity compared to those found in humans [56]. This new hypothesis sparked scientists to investigate this phenomenon at a molecular level (Figure 2). Ravasi et al. [57] investigated the genotype of human and non-human primates in Central Africa. Sequencing of the rDNA (ITS1-5.8S-1TS2) revealed two *Trichuris* genotypes that infect both humans and non-primates [57]. Ghai et al. [58] found similar results to Ravasi et al. [57], but three *Trichuris* genotypes were identified as circulating within human and non-human primates. Humans were infected with two genotypes: one genotype that was only common to human samples (Group 1), and another genotype that infected humans as well as non-human primates (black-and-white colobus (*Colobus guereza*), blue monkeys (*Cercopithecus mitis*), grey-cheeked mangabeys (*Lophocebus albigena*), l’hoest monkeys (*Cercopithecus lhoesti*), olive baboons (*Papio anubis*), red colobus (*Procolobus rufomitratus*), red-tailed guenons (*Cercopithecus ascanius*), and the chimpanzee (*Pan troglodytes*)) (Group 3). The intermediary group (Group 2) had a *Trichuris* genotype that affected non-human primates (black-and-white colobus (*Colobus guereza* and the red colobus (*Procolobus rufomitratus*) [58]. Furthermore, this new species of *Trichuris* was found in the Francois’ leaf monkey (*Presbytis francoisi*) and the *Colobus guereza kikuyensis* using mtDNA, rDNA, and morphometry [59,60].

#### 3.2.4. Molecular Identification *Trichuris* spp. in Rodents

*Trichuris* spp. has been found in domestic livestock and pets, but there are also species that are specific to rodents. The initial molecular research that was done on the *Trichuris* spp. present in rodents focused on European rodents [61]. *Trichuris muris* was identified in Murid rodents in Europe with the use of rDNA (ITS1-5.8S-ITS2). It was found that two lineages had occurred, due to geographical distribution. One was found in northern Spain to Denmark, and the other in the Southern Europe (Croatia, Romania, and Turkey) [61]. In recent years, several new species of *Trichuris arvicolae* have been found in Arvicolinae rodents using multi-local enzyme electrophoresis [62] and rDNA (ITS1-5.8S-ITS2) [63]. Further investigations were done in the phylogeographic analysis of *T. arvicolae* in Europe, using the mtDNA cytochrome subunit 1 gene (cox1) and rDNA (ITS1-5.8S-ITS2). Nuclear genetics (ITS1-5.8S-ITS2) suggest that *T. arvicolae* show two geographic and genetic lineages (Neoarctic and Palaearctic). Mitochondrial results gave further details into the Palaearctic region, giving three geographic and genetic lineages (Northern Europe, Southern and Eastern Europe, and Italy and France) [64]. 

Scientists also investigated *Trichuris* present in Sigmodontinae rodents in South America (Argentina). New species, such as *Trichuris novonae*, were identified based on morphological analysis [65]. Another species that was identified morphologically was *T. pardinasi* [64]. Based on molecular characteristics, using ITS2 (rDNA), a new species named *Trichuris bainae* was identified [66]. Molecular analysis using cox1 and mitochondrial cytochrome b (cob) on the *Trichuris* spp. found in Sigmodontinae rodents found three clades corresponding to three different species, which were *T. pardinasi*, *T. bainae*, and *T. navonae*) [67]. Further to this, *T. massoiai* was identified in *Holochilus chacarius* (Cricetidae: Sigmodontinae) using morphological mitochondrial (cox1 and cob) and nuclear (ITS2) markers [68].

Callejon et al. [41,69] investigated nuclear (18S, triose phosphate isomerase) and mitochondrial (cox1, cob1) genes from *Trichuris* spp. from nine various host species *(Colobus guereza kikuyuensis*, *Papio hamadryas*, *Homo sapiens*, *Sus scrofa domesticus*, *Capra hircus*, *Canis lupus familiaris*, *Bos taurus*, *Mus domesticus*, and *Myodes glareolus*) from Spain. The data show that *Trichuris* spp. could be divided in three clades: Clade 1 = *T. arvicolae*, *T. muris*, and *T.vulpis*; Clade 2 = *T. suis*, *T. colobae*, *T. trichiura*, and *T*. spp. ex *Papio hamadryas*; Clade 3 = *T. discolor*, *T. ovis*, and *T. skrjabini* [69].

### 3.3. Immunomodulatory Effect of Trichuris spp.

*Trichuris* spp. has been used in the treatment of gastrointestinal autoimmune diseases, such as inflammatory bowel disease, Crohn’s disease, and ulcerative colitis [70,71,72]. *Trichuris suis* (pig whipworm) had been experimentally given to humans with no overt sign of gastrointestinal illness. The eggs produced from the feces remained constant, and only a low percentage of these eggs embryonated in vitro [21]. Some authors also noted that treatment of patients with inflammatory bowel disease, ulcerative colitis, and Crohn’s disease with *Trichuris suis* showed improvement in gastrointestinal signs, and in the management of disease the subjects were given ova every three weeks [70,71,72]. Surprisingly, Kradin et al. [22] noted that a patient that underwent treatment for Crohn’s disease using *T. suis* had adult worms beneath the ileocecal mucosal epithelium. This case does raise concerns about persistent infection from *T. suis* in man [22].

Further work was done on the use of excretory secretory products of *T. suis* in rats [73]. The investigation of the use of excretory products of *T. suis* in swine epithelium cells was used as a model to be used in humans. It was noted that the excretory secretory products (ESPs) elicited the production of interleukin (IL)-6 and IL-10, which have been identified as anti-inflammatory cytokines that inhibit Th-1 responses. This proved that ESPs from *T. suis* have immunomodulatory effects and can be used as candidates in the treatment of inflammatory bowel disease [73]. The use of ESPs from *T. suis* may be safer than the actual treatment with ova. 

Subsequent research was done on the immunomodulatory and immunogenic effects of the proteins and ESPs of *Trichuris trichiura* and *Trichuris muris* [74,75,76]. Proteins were analyzed from adult worm extract and fragments of *T. trichiura*. These extracts and fragments were placed in cell cultures of human peripheral blood monocytes, and elicited the production of IL-10, IL-12, and TNF-α. Some fractions showed the inhibition of IL-5 production. The downregulation of IL-5 is a feature of a Th-2 response [74]. Santos et al. [74] concluded that protein fractions of *T. trichiura* can be used in the treatment and prevention of allergic and autoimmune diseases. Immunogenic research was also conducted on the ESPs of *T. muris*, and specific immunogenic proteins were identified. The structure of one such protein was Tm16, which was characterized and could be used in the production of a vaccine [75]. Shears et al. [76] noted that ESPs from *T. muris* elicited production of IL-9 and IL-13 when inoculated into rats. Eleven immunogenic proteins from the ESP of *T. muris* were also identified, and these could be used in the production of a vaccine [76]. Recent studies show that there is tremendous potential for *Trichuris* in human autoimmune disease, as well as vaccine development in rural countries where trichuriasis infections are prevalent. 

## 4. Domesticated Neo-Tropical Rodent

### 4.1. Guinea Pig (Cavia Porcellus)

The guinea pig is a domesticated rodent that is utilized for its meat in rural communities in South America and Africa. In rural communities, it provides food security and economic opportunity. These animals can be reared on local feed by-products and can produce four to nine offspring per female per year [5,6]. Several gastrointestinal parasites have been reported to inhabit these animals, with few reports on the clinical effect on these animals [3]. 

Several authors that have done work investigating the gastrointestinal parasites present in captive reared guinea pigs have failed to find *Trichuris* present [77,78,79,80,81,82]. Endoparasites of wild and captive reared guinea pigs were investigated in many countries, including the Democratic Republic of Congo [77], Cameroon [78], Iran [80], and Brazil [81,82]. In Peru, 3.5% of wild guinea pigs were infected with *Trichuris gracilis* [83], but in captivity infection rates of 5% [84] and 31% [85] were recorded. However, in captive reared guinea pigs present in Cameroon, 0.3% [86] and 3.3% [87] were positive for *Trichuris* spp. In Benin, guinea pigs reared in captivity using traditional and modern housing arrangements had an infection rate for *Trichuris* spp. of 11.18% [88] (Table 1). Infection with elevated levels of *Trichuris* in domesticated animals can lead to diarrhea, weight loss, enteritis, and colitis. 

The authors who failed to identify *Trichuris* in guinea pigs used the fecal floatation technique [77,78,79,80,81,82] (using sodium chloride and zinc sulphate solution), gross identification of adult worms, as well as a combination of both methods mentioned above [80]. *Trichuris gracilis* in some studies was identified by the gross identification of adult worms in wild animals and captive animals [83,84,85], but in Cameroon and Benin *Trichuris* spp. was identified using fecal floatation and fecal sedimentation [86,87,88,94]. The weights, clinical conditions, or pathological findings of guinea pigs infected with *Trichuris gracilis* was not described by investigators. In most cases of trichuriasis in the guinea pig, there were parasites (*Paraspidodera uncinata*, *Capillaria* spp., or *Trichostrongylus colubriformis*) that co-infected the hosts’ gastrointestinal tract. However, Garcia et al. [95] reported that 55% of the animals were infected only with *Trichuris* spp.

*Trichuris* spp. was identified in several countries, including those found in the African and South American continent. Guinea pigs that were wild and captive reared were both found to have *Trichuris* spp. However, the species of *Trichuris* was not identified in most cases, due to a lack of molecular techniques in the detection of this parasite. The genus *Trichuris* has zoonotic significance, since human and non-human primates are infected with *Trichuris trichiura* [54]. Future work should focus on molecular technique in the identification of parasites in the guinea pig and their potential immunoregulatory effects in experimental studies. 

### 4.2. Semi-Domesticated Neo-Tropical Rodents

#### 4.2.1. Agouti (*Dasyprocta leporina*)

The agouti is a robust rodent, with adults weighing 2–4 kg [8]. These animals are omnivorous [96], practice cecotrophy, and possess a large cecum [97]. Some authors have even classified these animals as opportunistic omnivores [98]. These animals have been successfully fed in captivity [89], and several endoparasites have been identified in this animal [4]. Parasites that have been found in the agouti seem to have no effect on the animals clinically or sub-clinically [4]. Animals that have parasites living within their digestive tract appear to be well-fleshed, with the absence of any gastrointestinal disturbances.

Early parasitic investigation done on the agouti using the morphological data of adult specimens in the digestive tract identified *Trichuris gracilis* var. *trinitatae* from wild agoutis in Trinidad [99]. Further to this initial work, Suepaul et al. [100] found *T. gracilis* var*. trinitatae* in hunted agouti in Trinidad, and Goncalves et al. [90] identified *T. gracilis* var*. trinitatae* in wild agouti in Brazil. In recent times, the identification of different *Trichuris* spp. using the morphological analysis of adult worms and eggs have proven to be inadequate [90]. In wild animals *Trichuris* spp. was identified in conjunction with other gastrointestinal helminths. However, authors have failed to record data on the health of the animals or the pathology of the gastrointestinal tract.

*Trichuris* spp. was also identified using fecal floatation techniques in wild and captive reared agoutis. Species identification was impossible with the use of fecal floatation. In Brazil, *Trichuris* spp. was identified in wild agoutis, with eggs having their characteristic bi-polar plugs [101]. Further research done in Trinidad identified *Trichuris* spp. in farmed agoutis [102]. *Trichuris* spp. was identified along with *Strongyloides* spp., *Eimeria* spp., and *Paraspidodera uncinata* [103]. Infected animals had an average fecal egg count of 2.2 × 10^2^_,_ and animals were in good body condition, with no gross pathological lesions and blood values within normal reference ranges [103,104].

The prevalence of *Trichuris* spp. in captive reared animals in Trinidad was 4.62% [102]. Suepaul et al. [100] obtained a higher prevalence of 53.95% in free range agoutis (Table 1). These studies are the only record in the literature that report the prevalence of *Trichuris* in the agouti. This shows the limited research done on trichuriasis in this neo-tropical rodent. In the agouti, there has been an absence of molecular identification of endoparasites, and in particular to *Trichuris*. The agouti has been grown in captivity, with close contact with humans and domesticated animals. Thus, species identification of the presence of this parasite is paramount. *Trichuris* appears to be ubiquitous in captive and wild environments of the agouti, and proper analysis of the effect of this parasite must be documented.

#### 4.2.2. Lappe (*Agouti paca*/*Cuniculus paca*)

The lappe is a robust neotropical rodent with adults weighing 4–8 kg [8]. These rodents practice cecotrophy and consume locally available fruits and crops [98]. The majority of parasitic investigations done on the lappe have focused on echinococcosis. This is because the lappe is the intermediate host for *Echinococcus* spp. [103,104,105,106,107], with the dog as the final host. Humans can become dead-end intermediate hosts following ingestion of eggs shed by dogs, but cannot become infected by meat or organ consumption.

In the lappe, adult *Trichuris* worms have been found in the cecum of the gastrointestinal tract [91], and eggs have been found in the feces using fecal floatation [92,108]. However, in the studies conducted, scientists failed to identify the species of *Trichuris* and its effect on the lappe. It is impossible to distinguish different species of *Trichuris* through the use of morphological identification of the adult worms or the eggs that are produced.

*Trichuris* spp. was found in both captive [105,106] and wild lappe populations [91]. *Trichuris* was found in conjunction with several endoparasites in the digestive tract, with no cases being reported of lappe infected with only *Trichuris* spp. The research work reported on the lappe was done in the neotropics in Mexico, Costa Rica, and Brazil. The prevalence of this disease varied between locations, with 10% of lappe in the study in Mexico being infected [92], while in Costa Rica, 2.13% (Table 1) of the sampled animals had *Trichuris* spp. [108]. In Brazil, animals had an average fecal egg load of 4.15 eggs per gram (EPG) [108]. 

The differences in prevalence of *Trichuris* seen between Costa Rica and Mexico can be due to firstly, the method of identification, with Ramirez-Herrera et al. [92] utilizing fecal floatation and Matamoros et al. [105] utilized gross identification of adult parasites. Secondly, these can be variations in the number of infected animals within the respective countries that contribute to the contamination of the environment with infective eggs. Thirdly, the environment in which the sampled animals inhabit may be different, with the lappes studied in Costa Rica being raised in the wild and lappes sampled in Mexico being maintained in a captive environment. Further research must be done to obtain the prevalence of trichuriasis in the lappes of various countries within the neo-tropical region. The species identification must also be performed in these investigations, as well as a comparison of prevalence between captive and wild populations, using molecular techniques for identification. 

#### 4.2.3. Capybara (*Hydrochoerus hydrochaeris*)

The capybara is the largest rodent in the world. Lall et al. [96] summarized these animals as semi-aquatic herbivorous rodents that practice cecotrophy. These animals are hindgut fermenters that possess a mucus trap separation mechanism [109]. Various parasites have been found in the capybara, but the majority of research views these animals as reservoirs for specific pathogens that have zoonotic potential or can cause disease in domestic livestock species.

The major parasites investigated have included helminths and protozoa. Helminths like *Fasciola hepatica* has been found in both capybara and cattle, but cause major disease problems in cattle [110,111,112]. Protozoan parasites of zoonotic importance reported in capybara include *Cryptosporidium parvum* [113]. Protozoan parasites of found in the capybara that can negatively affect livestock include *Eimeria* spp., *Eimeria ichiloensis*, *Eimeria boliviensis*, and *Eimeria trinidadensis* [114,115,116,117,118,119]. However, few reports have been made on the identification of *Trichuris* or the clinical effect of this pathogen.

Brazilian capybaras reared in captivity had *Trichuris* spp. in 50% of samples [117] (Table 1). *Trichuris* spp. was found in conjunction with several other parasites in the gastrointestinal tract. The effects of these endoparasites on the capybara have not been documented. Surprisingly, the capybara is the only rodent where molecular techniques have been used in the identification of *Trichuris* spp. Eberhardt et al. [93] identified a new species of *Trichuris* from capybara using molecular characteristics and phylogenetic relationships. The new species was identified as *Trichuris cutillasae*, and this was found in the cecum of capybara in Argentina. This new species has veterinary importance, and emphasizes the fact that further work has to be done on the genetic identification of *Trichuris* spp. in the capybara at different geographical locations in the neo-tropics. This new species must be investigated to provide clarity on the effect of this parasites on the health and performance of the capybara. Information on the parasitic load of *Trichuris cutillasae* in the capybara that will precipitate disease need to be investigated.

## 5. Conclusions

This review revealed that *Trichuris* has been found in the guinea pig (*C. porcellus*), agouti (*D. leporina*), lappe (*A. paca*/*C. paca*), and capybara (*H. hydrochaeris*). However, there is a dearth of information on the species of *Trichuris* that parasitize these animals. The capybara was the only rodent where some molecular techniques were used to identify a new species of *Trichuris*, named *T. cutillasae*. In most cases, *Trichuris* was found in combination with other endoparasites, and had a prevalence ranging from 4.62–53.85% in the agouti, 4.21–10.00% in the lappe, 50% in the capybara, and 1–31% in the guinea pig. 

## 6. Recommendations

Further work must be done on the molecular identification of various *Trichuris* spp. present in neo-tropical rodents, as well as the effect of *Trichuris* on the performance of agouti, lappe, capybara, and guinea pigs.

## Figures and Tables

**Table 1:** Prevalence of *Trichuris* spp. in different locations in neo-tropical rodents.

Species	Geographical Location	Environment	Sample Size (*n*)	Prevalence (%)	Reference
*Cavia porcellus*	Benin	Captive reared	18	2/18 (11.11)	[88]
*Cavia porcellus*	Peru	Captive reared	400	20/400 (5.00)	[84]
*Cavia porcellus*	Peru	Captive reared	100	31/100 (31.00)	[85]
*Cavia porcellus*	Cameroon	Captive reared	397	4/397 (1.00)	[86]
*Cavia porcellus*	Cameroon	Captive reared	300	10 (3.30)	[87]
*Cavia aperera*	Peru	Free range	143	5/143 (3.50)	[83]
*Dasyprocta leporina*	Trinidad	Free range	13	11/13 (53.85)	[89]
*Dasyprocta leporina*	Trinidad	Captive reared	65	3/65 (4.62)	[90]
*Agouti paca*	Costa Rica	Captive reared	140	3/140 (2.41)	[91]
*Agouti paca*	Mexico	Captive reared	10	1/10 (10.00)	[92]
*Hyrdochoerus hydrochaeris*	Brazil	Captive reared	24	12/24 (50.00)	[93]

## Data Availability

All data used are presented in the document.
